# Integrating *Escherichia coli* Antimicrobial Susceptibility Data from Multiple Surveillance Programs

**DOI:** 10.3201/eid1106.041160

**Published:** 2005-06

**Authors:** John M. Stelling, Karin Travers, Ronald N. Jones, Philip J. Turner, Thomas F. O'Brien, Stuart B. Levy

**Affiliations:** * Alliance for the Prudent Use of Antibiotics, Boston, Massachusetts, USA;; † Brigham and Women's Hospital, Boston, Massachusetts, USA;; ‡ The Jones Group, North Liberty, Iowa, USA;; § Tufts University, Boston, Massachusetts, USA;; ¶ AstraZeneca, Macclesfield, Cheshire, United Kingdom

**Keywords:** antimicrobial resistance, Escherichia coli, antimicrobial susceptibility, drug resistance surveillance

## Abstract

Collaboration between networks presents opportunities to increase analytical power and cross-validate findings. Multivariate analyses of 2 large, international datasets (MYSTIC and SENTRY) from the Global Advisory on Antibiotic Resistance Data program explored temporal, geographic, and demographic trends in *Escherichia coli* resistance from 1997 to 2001. Elevated rates of nonsusceptibility were seen in Latin America, southern Europe, and the western Pacific, and lower rates were seen in North America. For most antimicrobial drugs considered, nonsusceptibility was higher in isolates from men, older patients, and intensive care unit patients. Nonsusceptibility to ciprofloxacin was higher in younger patients, rose with time, and was not associated with intensive care unit status. In univariate analyses, estimates of nonsusceptibility from MYSTIC were consistently higher than those from SENTRY, but these differences disappeared in multivariate analyses, which supports the epidemiologic relevance of findings from the 2 programs, despite differences in surveillance strategies.

The World Health Organization (WHO) highlights the establishment of "effective, epidemiologically sound surveillance of antimicrobial resistance among common pathogens in the community, hospitals, and other health care facilities" as 1 of 2 fundamental public health priorities in efforts to confront antimicrobial drug–resistant organisms (1). At present, most data published in the international literature on antimicrobial resistance are derived from short-term surveys of specific organisms and agents in defined areas. Consequences of this nonsystematic, discontinuous approach are the inability to establish meaningful baseline trends; low sensitivity in detecting new threats; inadequate information to evaluate interventions; and lack of data on organisms, antimicrobial drugs, and patient populations not included in the surveys.

Surveillance groups must coordinate efforts to provide the broadest set of data to policymakers and researchers and to assess the reliability of findings from individual systems. Recognizing the urgency of the problem and the value of joint surveillance collaborations, the Alliance for the Prudent Use of Antibiotics (APUA), a nonprofit organization, established the Global Advisory for Antibiotic Resistance Data (GAARD) (2) in 1999 to involve several of the world's largest multinational enterprises tracking global trends in resistance as well as the Centers for Disease Control and Prevention, WHO, and the WHO Collaborating Centre for Surveillance of Antimicrobial Resistance, which serve in advisory roles. Currently, AstraZeneca International (supporting the Meropenem Yearly Susceptibility Test Information Collection [MYSTIC] surveillance project), Bayer AG (TARGETed), Bristol-Myers Squibb Company (SENTRY), GlaxoSmithKline (Alexander Project), and Ortho-McNeil Pharmaceuticals (TRUST) work with APUA to provide data for GAARD studies. In 2002, data were collected from then-participating GAARD members on *Streptococcus pneumoniae* (3), *Haemophilus influenzae* (4), and *Escherichia coli*. The focus of this article is the analysis of submitted *E. coli* results from GAARD-participating systems tracking *E. coli* at that time, i.e., MYSTIC and SENTRY.

*E. coli* is the most common cause of infections by gram-negative bacilli (5) and the bacterial organism most often isolated from blood cultures (6–9). It is a frequent cause of outpatient urinary tract infections in women worldwide, of hospitalization due to pyelonephritis and septicemia, and of nosocomial infections among hospitalized patients. Meningitis caused by *E. coli* in neonates is frequently fatal. Resistance to recommended first- and second-line agents, such as penicillins, cephalosporins, sulfa drugs (5,7,10), and fluoroquinolones (11,12) is high in many countries and is commonly associated with treatment failure (13,14).

## Methods

Antimicrobial susceptibility data on *E. coli* collected by the MYSTIC and SENTRY systems were forwarded to GAARD coordinators at APUA for descriptive and inferential analysis of temporal, demographic, and geographic trends. MYSTIC was launched by AstraZeneca in 1997 to study bacterial resistance in specialist and general hospital units in hospitals using meropenem (15). At present, 52 sites from 19 countries are contributing results. Each center isolates up to 100 gram-positive and 100 gram-negative aerobic bacteria per year from routine diagnostic samples from hospitalized patients, excluding repeat patient isolates. Antimicrobial susceptibility tests are performed by broth microdilution by using NCCLS reference methods (16) either on-site (for non-US laboratories) or by a reference laboratory (for US participants). More than 9,000 isolates are processed annually, with at least 9 antimicrobial drugs tested per strain.

Bristol-Myers Squibb established the SENTRY program in 1997 as a global program for the surveillance of resistance in bacterial and fungal populations (17). SENTRY has expanded from 75 sites in 1997 to 94 laboratories in 35 countries in 2003. Bacterial isolates are obtained from diagnostic specimens taken in the course of routine clinical management of both hospitalized and community patients. Each site collects a defined number of consecutively identified strains within a number of distinct protocols, e.g., blood isolates, urine isolates, and respiratory isolates, excluding repeat patient isolates. Strains, including basic patient demographic data, are shipped to a coordinating laboratory for centralized identification and susceptibility testing by broth microdilution panels according to NCCLS reference guidelines (16). Forty-five to 50 antimicrobial drugs are monitored each year, with ≈30 tested per strain; >200,000 strains are processed annually.

### Available Data

**Figure 1:**
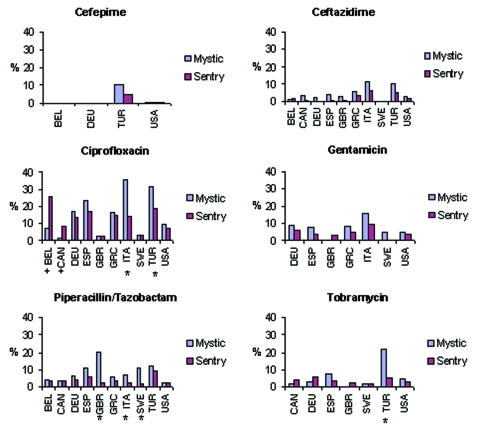
Comparison of MYSTIC and SENTRY rates of *Escherichia coli* nonsusceptibility rates in 2001 to antimicrobial drugs tested by both networks. Significant findings are indicated with an asterisk where the MYSTIC estimate is higher than the SENTRY result and with a plus sign when the SENTRY estimate is higher. Country codes are the official 3-letter codes designated by the International Organization for Standardization: BEL, Belgium; CAN, Canada; DEU, Germany; ESP, Spain; GBR, United Kingdom; GRC, Greece; ITA, Italy; SWE, Sweden; TUR, Turkey; and USA, United States.

Data on *E. coli* from 1997 to 2001 were available from 24 countries from the MYSTIC program (4,818 isolates) and 34 countries from SENTRY (14,819 isolates). Because 20 countries are tested by both systems, this figure represents 38 countries, as shown in Table 1. Numbers in the table indicate the number of centers that contributed data at any point during the 5-year period. Descriptive analyses and multivariate regressions included data from all countries, except when data were insufficient (defined as <30 isolates in 2000 and 2001) (18): MYSTIC data from Bulgaria, Malta, Russia, Switzerland, Hong Kong, and Thailand and SENTRY data from Austria, the Netherlands, Portugal, Russia, Mexico, Uruguay, and China. Data from both networks were available for 16 countries, but direct univariate comparisons of findings between the 2 networks were limited to the 10 countries, shown in Figure 1, with at least 20 isolates in each of the years displayed. These 10 "comparison" countries, principally representing North America and Europe, are Belgium, Canada, Germany, Greece, Italy, Spain, Sweden, Turkey, the United Kingdom, and the United States. The United States provided 17% of the MYSTIC isolates and 46% of the SENTRY isolates.

**Table 1:** Countries participating in the MYSTIC and SENTRY programs*

System	North America	Latin America	Northern Europe	Southern Europe + South Africa	Western Pacific
MYSTIC (24 countries)	Canada (14, 97), United States (18, 816)	Argentina (3, 41), Brazil (3, 75), Colombia (1, 20), Mexico (4, 170)	Belgium (9, 572), Czech Republic (1, 90), Germany (7, 668), Poland (1, 70), Russia (1, 7),† Sweden (3, 153),† Switzerland (1, 40),† United Kingdom (8, 294)	Bulgaria (1, 10),† Greece (2, 37), Israel (1, 96), Italy (5, 369), Malta (1, 11),† Spain (5, 517), Turkey (9, 529)	Australia (1, 46), Hong Kong (1, 20),† Thailand (1, 70)†
Total (101 sites, 4,818 isolates)	32 sites, 913 isolates	11 sites, 306 isolates	31 sites, 1,894 isolates	24 sites, 1,569 isolates	3 sites, 136 isolates
SENTRY (34 countries)	Canada (8, 1,334), United States (36, 5,438)	Argentina (2, 282), Brazil (5, 488), Chile (2, 610), Colombia (1, 181), Mexico (3, 166),† Uruguay (1, 17),† Venezuela (1, 72)	Austria (1, 105),† Belgium (1, 171), Germany (6, 440), Ireland (1, 52), Netherlands (1, 107),† Poland (1, 141), Russia (1, 6),† Sweden (1, 112), Switzerland (1, 380), United Kingdom (1, 260)	France (9, 1,086), Greece (1, 212), Israel (1, 128), Italy (4, 431), Portugal (1, 91),† South Africa (1, 76), Spain (3, 1,007), Turkey (3, 217)	Australia (4, 480), China (3, 62),† Hong Kong (1, 228), Japan (3, 93), Philippines (1, 130), Singapore (2, 118), Taiwan (3, 98)
Total (114 sites, 14,819 isolates)	44 sites, 6,772 isolates	15 sites, 1,816 isolates	15 sites, 1,774 isolates	23 sites, 3,248 isolates	17 sites, 1,209 isolates

*The number of participating centers at any point from 1997 to 2001 and number of isolates by country are indicated in parentheses. †Countries excluded from analyses for insufficient data, as defined in the text.

### Antimicrobial Drugs

For *E. coli* in the MYSTIC project, either 12 (United States isolates) or 11 (non-US isolates) antimicrobial drugs were tested. In SENTRY, 26 antimicrobial drugs were examined. The following 8 compounds were tested by both programs and will be referred to as the core antimicrobial agents for comparisons between the 2 networks: cefepime, ceftazidime, ciprofloxacin, gentamicin, imipenem, meropenem, piperacillin/tazobactam, and tobramycin. Because a primary objective of this study is to highlight the value in contrasting findings from different surveillance programs, most subsequent regression analyses will focus on these 8 agents.

With the exception of ciprofloxacin, these compounds are primarily administered as second-line therapy to hospitalized patients and not routinely to outpatients. Because monitoring resistance to first-line agents is essential to guide empiric treatment decisions, data from the SENTRY network are also presented for the following compounds not tested by MYSTIC laboratories: amoxicillin/clavulanic acid, ampicillin, nalidixic acid, nitrofurantoin, tetracycline, and trimethoprim/sulfamethoxazole.

### Data Analysis

Similar demographic data were available from both systems and included patient country, age, and sex; intensive care unit (ICU) or non-ICU location; and specimen type. Susceptibility test data were recorded as MIC values. Resistant, intermediate, and susceptible categories were interpreted according to 2003 NCCLS guidelines (19). During the period studied, NCCLS breakpoints did not change for the drugs studied. Strains with a clinical interpretation of resistant or intermediate were considered nonsusceptible in further analyses.

Available data on *E. coli* from 1997 through 2001 were sent in Microsoft Excel (Microsoft Corp., Redmond, WA, USA) format by MYSTIC and SENTRY coordinators to APUA for analysis. For descriptive data analysis, files were imported into WHONET 5.2 (World Health Organization, Geneva, Switzerland) (20). Univariate analyses by chi-square testing and multivariate logistic regressions were carried out with Intercooled STATA v. 7 (StataCorp LP, College Station, TX, USA), with null hypotheses rejected for values of p<0.05 and without correction for multiple comparisons. Age was categorized in 10-year intervals, and countries were categorized by geographic region defined in Table 1.

## Results

### Univariate Comparison of Surveillance Networks

A comparison of the MYSTIC and SENTRY results for 2001 is shown in Figure 1 for the 10 comparison countries. Excluding ciprofloxacin, resistance rates were ≤10% in 2001 for the core antimicrobial drugs among the comparison countries, with the following exceptions: ceftazidime (11.4%) and gentamicin (15.7%) in Italy (MYSTIC); tobramycin (21.9%) in Turkey (MYSTIC); and piperacillin/tazobactam in Spain (10.8%), Sweden (10.9%), Turkey (11.9%), and the United Kingdom (20.9%) (MYSTIC). No isolates confirmed resistant to meropenem or imipenem were found by SENTRY. In the MYSTIC dataset, 2 isolates (from Mexico and Turkey) were found to be nonsusceptible to meropenem and 23 (from Belgium, Brazil, Germany, Mexico, Malta, Turkey, and the United Kingdom) to imipenem. As part of an ongoing protocol for quality assurance, several of these isolates were subsequently confirmed through centralized testing.

Nonsusceptibility estimates in MYSTIC data were consistently higher than in SENTRY. For the 2001 data, country-specific comparisons of MYSTIC to SENTRY nonsusceptibility rates were examined for each antimicrobial drug. From the 46 possible comparisons, MYSTIC estimates were higher than in SENTRY 37 times (80.4%, sign test p<0.001). Excluding comparisons in which either rate was equal to 0%, MYSTIC estimates were on average 2.2 times higher than SENTRY values. Subsequent analysis suggests that the principal contributor to the differences between the surveillance systems would be the higher proportion of ICU patients in MYSTIC (38.0%, n = 1,468) than in SENTRY (19.5%, n = 2,642). Significant differences are depicted in Figure 1.

### Univariate Temporal Trends in *E. coli* Nonsusceptibility

**Figure 2:**
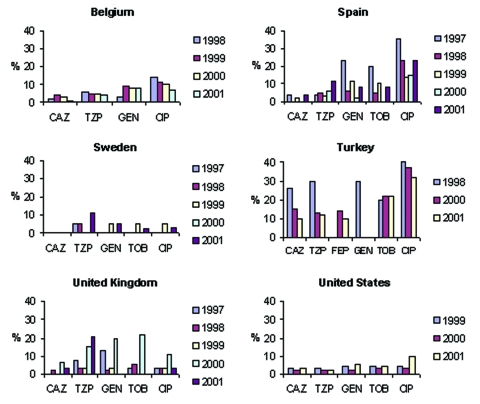
MYSTIC results for comparison countries. Annual nonsusceptibility rates of *Escherichia coli* isolates, 1997–2001. p<0.05. CAZ, ceftazidime; TZP, piperacillin/tazobactam; GEN, gentamicin; CIP, ciprofloxacin; TOB, tobramycin; FEP, cefepime.

**Figure 3:**
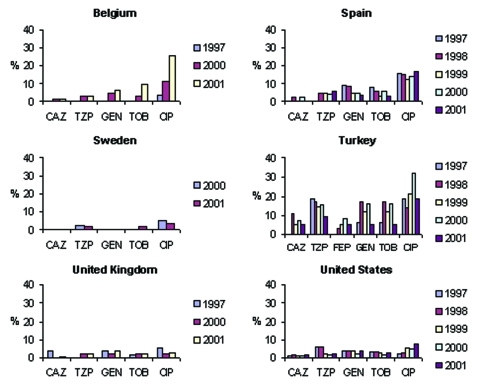
SENTRY results for antimicrobial agents tested in common with MYSTIC. Annual nonsusceptibility rates of *Escherichia coli*, 1997–2001. p<0.05. CAZ, ceftazidime; TZP, piperacillin/tazobactam; GEN, gentamicin; CIP, ciprofloxacin; TOB, tobramycin; FEP, cefepime.

Temporal trends from several of the comparison countries are shown in Figures 2, Figure 3, and 4. With the exception of ciprofloxacin, the antimicrobial drugs tested by both systems are principally reserved for intravenous use in hospitalized patients in most countries, and nonsusceptibility rates for these second-line agents were low worldwide, with some exceptions. Countries with nonsusceptibility rates ≥20% to at least 3 of the core agents by at least 1 of the systems in 2000 or 2001 include Israel, Poland, Mexico, Venezuela, Hong Kong, and the Philippines.

Significant trends (chi-square test for trend without correction for multiple comparisons, p<0.05) evident in the SENTRY dataset include increasing susceptibility to piperacillin/tazobactam in Argentina, Australia, Brazil, Chile, Israel, and the Philippines; increasing susceptibility to cefepime in Argentina and Brazil but decreasing susceptibility in Israel; increasing susceptibility to gentamicin in Brazil and Hong Kong; increasing susceptibility to tobramycin in Australia and Brazil; and decreasing susceptibility to ciprofloxacin in Belgium, Canada, Colombia, and the United States. MYSTIC data showed a significant decreasing trend in nonsusceptibility to ciprofloxacin in Belgium; susceptibility to piperacillin/tazobactam decreased in the United Kingdom; and susceptibility to gentamicin and tobramycin decreased in Israel.

**Figure 4:**
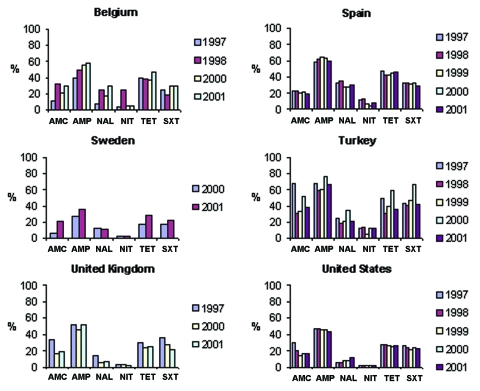
SENTRY results for supplemental antimicrobial drugs tested only by SENTRY. Annual nonsusceptibility rates of *Escherichia coli*, 1997–2001. AMC, amoxicillin/clavulanic acid; NAL, nalidixic acid; TET, tetracycline.

Figure 4 shows trends in nonsusceptibility data in comparison countries for a number of antimicrobial drugs tested only by the SENTRY system commonly prescribed in the outpatient setting. Nonsusceptibility for multiple first-line agents was high (approaching or exceeding 50%) in South Africa, Turkey, Brazil, Chile, Colombia, Venezuela, Hong Kong, the Philippines, Singapore, and Taiwan. Noteworthy trends (p<0.05, chi-square for trends without correction for multiple comparisons) were noted for a number of these agents. Increasing susceptibility to amoxicillin/clavulanic acid was seen in Argentina, Brazil, Canada, Chile, Italy, the United Kingdom, and the United States. Increasing susceptibility to trimethoprim/sulfamethoxazole was seen in Singapore, Chile, Australia, the United States, and Italy, but decreasing susceptibility was seen in Germany; susceptibility to ampicillin decreased in Germany, Colombia, and the Philippines but increased in Chile. Susceptibility to nalidixic acid decreased in Belgium, Canada, Germany, and the United States; susceptibility to nitrofurantoin increased in Canada, Spain, and Chile. Susceptibility to tetracycline increased in Italy and the United Kingdom but decreased in Germany.

### Multivariate Trends in *E. coli* Nonsusceptibility

Multivariate logistic regression was performed to simultaneously control for the effect of potentially confounding variables on nonsusceptibility rates. Independent variables included region, age group, sex, specimen year, ICU specimen source, and surveillance system. Table 2 highlights the significant factors. Because of the rarity of meropenem- and imipenem-resistant isolates in the database, these agents were not studied by logistic regression.

**Table 2:** Odds ratios (OR) and 95% confidence intervals (CI) from multivariate analysis of risk factors for nonsusceptibility in *Escherichia coli*, 1997–2001*

Drug	Factor	OR (95% CI)	p value
Cefepime (18,239 isolates)	Southern Europe	2.23 (1.08–4.69)	0.034
	Latin America	4.82 (2.58–9.012)	<0.001
	North America	0.35 (0.16–0.76)	0.008
	Western Pacific	6.39 (1.98–20.56)	0.002
	Age group	1.74 (1.09–2.79)	0.021
	Intensive care unit	2.84 (1.93–4.17)	<0.001
Ceftazidime (19,404 isolates)	Southern Europe	2.20 (1.20–4.06)	0.011
	Latin America	4.79 (2.83–8.12)	<0.001
	Age group	1.94 (1.38–2.75)	<0.001
	Intensive care unit	2.25 (1.69–3.01)	<0.001
Ciprofloxacin (19,320 isolates)	Northern Europe	1.62 (1.18–2.23)	0.003
	Southern Europe	2.99 (2.27–3.93)	<0.001
	Latin America	3.76 (2.93–4.84)	<0.001
	North America	0.77 (0.60–0.99)	0.046
	Western Pacific	3.07 (1.63–5.76)	<0.001
	Male	1.46 (1.26–1.68)	<0.001
	Age group	0.39 (0.29–0.52)	<0.001
	Year	1.14 (1.07–1.21)	<0.001
Gentamicin (18,773 isolates)	Latin America	2.44 (1.86–3.20)	<0.001
	North America	0.74 (0.56–0.97)	0.027
	Western Pacific	4.64 (2.66–8.09)	<0.001
	Male	1.28 (1.09–1.52)	0.004
	Age group	1.47 (1.15–1.88)	0.002
	Intensive care unit	1.23 (1.01–1.51)	0.042
Piperacillin/tazobactam (19,261 isolates)	Southern Europe	2.01 (1.38–2.92)	<0.001
	Latin America	2.18 (1.60–2.96)	<0.001
	Western Pacific	2.11 (1.01–4.40)	0.046
	North America	0.73 (0.54–0.99)	0.040
	Male	1.33 (1.09–1.61)	0.004
	Year	0.74 (0.68–0.81)	<0.001
	Intensive care unit	1.51 (1.24–1.92)	<0.001
Tobramycin (18,416 isolates)	Southern Europe	1.43 (1.00–2.05)	0.047
	Latin America	3.09 (2.31–4.13)	<0.001
	Western Pacific	3.42 (1.77–6.63)	<0.001
	North America	0.70 (0.52–0.94)	0.019
	Male	1.31 (1.10–1.57)	0.003
	Age group	1.66 (1.30–2.13)	<0.001
	Intensive care unit	1.37 (1.11–1.69)	0.003

*Logistic regression models simultaneously controlled for geographic region, age categories, sex, intensive care unit status, year of specimen, and reporting system. Only significant associations are presented. No significant relationships between nonsusceptibility and reporting system (MYSTIC vs. SENTRY) were found.

Certain regions (southern Europe, Latin America, and western Pacific), male sex, older age, and ICU isolates were consistently (for at least 4 of the 6 drugs) associated with higher nonsusceptibility rates. North American isolates had lower nonsusceptibility rates (for 5 of the 6 drugs), while isolates from northern Europe had higher rates only for ciprofloxacin. Significant temporal trends were identified only with ciprofloxacin (decreased susceptibility over time, odds ratio [OR] 1.14, 95% confidence interval [CI] 1.07–1.21, p<0.001) and piperacillin/tazobactam (increased susceptibility, OR 0.74, 95% CI 0.68–0.81, p<0.001). For ciprofloxacin, in contrast to findings with other agents, younger age was associated with a higher risk for nonsusceptibility (OR 0.39, 95% CI 0.29–0.52, p<0.001), and nonsusceptibility was not associated with ICU status. An important finding of the multivariate analysis is that the surveillance system (MYSTIC vs. SENTRY) was not associated with nonsusceptibility for any of the compounds, in contrast to the findings of the univariate analyses.

## Discussion

Through integrated analysis of data from multiple sources, the GAARD project seeks to realize a number of benefits: 1) increased statistical power in detecting evolutionary events of public health importance and elucidating risk factors for resistance emergence and spread; 2) greater geographic, demographic, and temporal coverage of bacterial populations than is possible under any single system with limited resources; and 3) cross-validation of findings from complementary data sources with distinct strategies for site recruitment, patient identification, specimen collection, and laboratory testing, which should prompt deeper investigation of seemingly discordant findings (21).

For countries in which a direct comparison of results from the 2 systems was possible, resistance frequencies from MYSTIC were typically higher than from SENTRY. In only 2 instances were higher SENTRY estimates significant (ciprofloxacin in Belgium and Canada). Observation of such incongruent findings should prompt a focused review for possible rationales, such as laboratory testing errors, differences among patient populations sampled, criteria for specimen selection, antimicrobial use patterns, or local outbreaks of resistant organisms. Because SENTRY estimates for Belgium reflect the experience of a single institution while MYSTIC data include results from 9 sites, the MYSTIC results may better reflect the situation in that country.

One of the most substantial findings of the multivariate analysis is that the surveillance system was not associated with nonsusceptibility in any of these compounds, in contrast to the findings of the univariate analyses. Thus, the finding that MYSTIC estimates of nonsusceptibility were consistently higher than SENTRY isolates in paired comparisons may be completely attributable to differences in the demographics of the patient subpopulations sampled. In this study, the principal contributor identified was the proportion of ICU patients represented in the 2 systems. Such findings should increase confidence in the reliability and validity of findings reported separately from the 2 programs. The observation of consistent differences in uncontrolled comparisons of results between systems also highlights the importance of including relevant demographic information in reports on antimicrobial susceptibility rates.

An arbitrary categorization of countries into relatively low, medium, and high resistance is shown in Table 3 for a few key first- and second-line antimicrobial drugs used to treat *E. coli* infections. The intervals indicated were selected to provide some degree of separation between groups of countries using the observed estimates and should not be interpreted as having a direct clinical implication for therapy decisions. The high rates of resistance to both ampicillin and trimethoprim/sulfamethoxazole in many countries observed in this study should prompt close review of treatment success rates in settings in which they are commonly used in empiric therapy (22).

**Table 3:** Nonsusceptibility rates of *Escherichia coli* by region, 2001*

Drug	North America	Latin America	Northern Europe	Southern Europe + South Africa	Western Pacific
Ampicillin					
20%–40%	Canada (35%)		Sweden (31%)	Italy (40%)	Japan (30%)
40%–60%	United States (44%)	Argentina, Brazil, Chile, Venezuela (54%–57%)	Belgium, France, Germany, Ireland, Switzerland, United Kingdom (46%–57%)	Greece (51%)	Australia, Singapore (50%–54%)
>60%		Colombia, Mexico (71%–76%)	Poland (62%–84%)	Israel, South Africa, Spain, Turkey (62%–84%)	Hong Kong, Philippines, Taiwan (64%–82%)
Trimethoprim/sulfamethoxazole					
0%–20%				Italy (19%)	Australia, Japan (11%–17%)
20%–40%	Canada, United States (20%–23%)	Argentina, Chile (28%–39%)	Belgium, Ireland, Poland, Sweden, Switzerland, United Kingdom (20%–31%)	France, Greece, Spain (20%–31%)	
40%–60%		Brazil, Colombia, Mexico, Venezuela (51%–57%)	Germany (40%)	Israel, South Africa, Turkey (42%–59%)	Hong Kong, Philippines, Singapore, Taiwan (40%–60%)
Ceftazidime					
≤5%	Canada, United States (MYS 3%, SEN 1%–2%)	Brazil, Chile (SEN 2%–4%)	Belgium, Czech Republic, Germany, Ireland, Poland, Sweden, Switzerland, United Kingdom (MYS 0%–3%, SEN 0%–3%)	Greece (SEN), France, South Africa Spain, Turkey (SEN) (MYS 4%, SEN 0%–5%)	Australia, Hong Kong, Japan, Singapore (MYS 0%, SEN 2%–3%)
>5%		Argentina, Colombia, Mexico, Venezuela (MYS 7%–13%, SEN 6%–11%)		Greece (MYS), Israel, Italy, Turkey (MYS) (MYS 6%–11%, SEN 6%–8%)	Philippines, Taiwan, Thailand (MYS 19%, SEN 6%)
Ciprofloxacin					
≤10%	United States, Canada (MYS 2%–10%, SEN 7%–9%)	Argentina (MYS), Brazil (SEN) (MYS 4%, SEN 10%)	Belgium (MYS), Czech Republic, Ireland, Poland, Sweden, Switzerland, United Kingdom (MYS 0%–7%, SEN 0%–9%)	France, South Africa (SEN 2%–6%)	Australia, Japan (MYS 0%, SEN 0%–2%)
>10%		Argentina (SEN), Brazil (MYS), Chile, Colombia, Mexico, Venezuela (MYS 14%–17%, SEN 12%–26%)	Belgium (SEN), Germany (MYS 18%, SEN 14%–26%)	Greece, Israel, Italy, Spain, Turkey (MYS 14%–39%, SEN 14%–30%)	Hong Kong, Philippines, Singapore, Taiwan (SEN 12%–31%)

*For countries with <30 isolates in 2001, data from 2000 and 2001 were combined. Ranges of nonsusceptibility rates are indicated in parentheses. For ampicillin and trimethoprim/sulfamethoxazole, data are only available from the SENTRY system. MYS, MYSTIC; SEN, SENTRY.

The use of surveillance data to guide antimicrobial therapy guidelines is a complicated issue that must address the constraints of available resources and therapeutic alternatives, local resistance and antimicrobial use patterns, and potential epidemiologic biases in available data. A number of studies have addressed empiric and quantitative approaches for using surveillance data in treatment guidelines for urinary tract infections and pyelonephritis, including cost-effectiveness studies and establishing resistance thresholds to guide therapy decisions (23–27).

Several significant results were noted in the univariate analyses of temporal trends. Such changes over time could be due to real shifts in the bacterial populations, changes in the number or type of participating institutions, changes in specimen collection practices, or spurious correlations, as no statistical corrections were made for multiple comparisons. The significant decrease to 4 or more agents in Brazil, Chile, and Italy in particular is worth highlighting for further exploration; Chile has successfully implemented and enforced new national legislation banning the sale of antimicrobial drugs without a prescription since 1999, and this legislation has produced substantial reductions in total antimicrobial drug use in the country (28).

Significant findings from the multivariate analysis of core antimicrobial drugs were mentioned above: higher rates of nonsusceptibility in isolates from ICU patients, older patients, and male patients and in isolates from Latin America, the western Pacific, and southern Europe. When all other variables were controlled for, nonsusceptibility to ciprofloxacin showed a statistical increase in over time, while nonsusceptibility to piperacillin/tazobactam decreased. This decrease in nonsusceptibility to piperacillin/tazobactam was significant in 11 countries in univariate analyses and merits further investigation into contributory factors. While temporal trends in the multivariate analysis may reflect, to some degree, the high proportion of US isolates in the SENTRY database, demographic characteristics of SENTRY isolates within and outside the United States were comparable, with only a small but significant difference seen for sex (44.2% [n = 1,058] male in the United States vs. 48.1% [n = 2,331] male outside the United States for 2001 data, p = 0.034).

The higher rate of nonsusceptibility among isolates from male patients has been previously noted for ciprofloxacin resistance (10,12,29) and ascribed to epidemiologic differences between men and women with *E. coli* infections. Urinary tract infections in male patients are more frequently complicated or healthcare-associated than those in the typical female patient, and infection in men may be associated with higher rates of previous antimicrobial drug usage and time in the hospital setting (29).

The finding of higher resistance in isolates from ICU patients to most agents is not unexpected, given the high selection pressure exerted by intensive antimicrobial use in this setting and the ease of transmission of resistant pathogens on the hands of healthcare workers. The observation that ICU isolates did not have higher rates of resistance to ciprofloxacin, most frequently used in the outpatient setting, suggests that risk factors for ciprofloxacin resistance are distinct from those of the other, principally second-line, agents studied. This dichotomy was also observed with respect to age. For ciprofloxacin, in contrast to the other core antimicrobial drugs, older age was associated with a significant protective effect, i.e., lower nonsusceptibility (OR 0.39, 95% CI 0.29–0.52, p<0.001), than seen in younger patients. One hypothesis holds that resistance in certain antimicrobial drugs, such as intravenous or second-line agents, is more closely associated with patterns of prescribing in hospitals and in older patients, while resistance in others, such as ciprofloxacin, is more correlated with patterns of antimicrobial drug use in the community. This hypothesis merits further investigation in a variety of geographic and clinical settings (30,31). Given the ubiquity of *E. coli* as a commensal pathogen in the human gut and in animal populations, resistance in *E. coli* may be a sensitive indicator of distinct therapeutic and nontherapeutic, appropriate and inappropriate uses of antimicrobial drugs (32). Another APUA-coordinated project, Reservoirs on Antibiotic Resistance, is a 5-year scientific collaboration that addresses this issue by exploring the movement of resistance determinants within commensal bacterial populations and between commensals and human pathogens (33).

Both the MYSTIC and SENTRY surveillance networks rely on routinely generated test results, a strategy with advantages over purely research-oriented, resource-intensive special surveys. These advantages include sustainability, more complete organism and geographic coverage, monitoring of baseline trends, infection control alerts, and outbreak detection. However, potential biases may be introduced that must be considered, such as selectively testing patients whose infections did not respond to treatment or who had more severe disease. Such biases may be amplified in the outpatient setting and in low-resource countries where treatment is frequently empiric with limited diagnostic testing. Results from routinely generated sample collections could usefully be compared to findings from periodic validation surveys in which greater resources are expended in identifying and testing representative patient populations (34–36).

With antimicrobial resistance continuing to evolve and present a global public health challenge, appropriately designed and implemented surveillance systems are a priority. Collaboration among existing surveillance systems can improve the quality, breadth, and impact of data for guiding and evaluating clinical and public health policy.
